# Overcoming barriers to equality, diversity, inclusivity, and sense of belonging in healthcare education: the Underrepresented Groups’ Experiences in Osteopathic Training (UrGEnT) mixed methods study

**DOI:** 10.1186/s12909-024-05404-3

**Published:** 2024-04-26

**Authors:** Jerry Draper-Rodi, Hilary Abbey, John Hammond, Oliver P. Thomson, Kevin Brownhill, Andrew MacMillan, Yinka Fabusuyi, Steven Vogel

**Affiliations:** 1 University College of Osteopathy, 275 Borough High Street, SE1 1JE London, UK; 2 National Council for Osteopathic Research, 275 Borough High Street, SE1 1JE London, UK; 3 School of Allied and Public Health Professions, North Holmes Road, CT1 1QU Canterbury, Kent, UK; 4 University of Portsmouth, University House, Winston Churchill Ave, PO1 2UP Hampshire, Portsmouth, England

**Keywords:** Underrepresented, Oppressed, Social Justice, Equity diversity and inclusion, Transformative action research, Osteopathic undergraduate education, Women, Disability, LGBTQIA+, Ethnic minorities

## Abstract

**Background:**

Individuals from minority groups have historically faced social injustices. Those from underrepresented groups have been less likely to access both healthcare services and higher education. Little is known about the experiences of underrepresented students during their undergraduate studies in osteopathy in the UK. The aim of this project was to explore awareness of cultural diversity and beliefs about patients from underrepresented groups in current osteopathic educational environments and evaluate students’ preparedness to manage patients from diverse groups. The project also aimed to investigate the educational experiences of students from underrepresented backgrounds during their training and their opinions on changes that could support better levels of recruitment and achievement. The findings were discussed with stakeholders in interactive workshops with the aim to develop recommendations for action and change.

**Methods:**

A transformative action research paradigm informed this mixed methods project. It included: 1/ a survey of students from all seven osteopathic educational providers in the UK using the Multidimensional Cultural Humility Scale (MCHS); 2/ a series of focus groups with students from underrepresented groups (women, students with disabilities, students from minority ethnic backgrounds, and students identifying as LGBTQIA+); and 3/ a workshop forum to discuss findings.

**Results:**

A total of 202 participants completed the MCHS and demographic questionnaire and seven focus groups were conducted. A model was developed to describe participants’ training experiences comprising two main themes: institutional contextual obstacles (with four sub-themes) and underrepresented students’ conceptual understanding of Equity, Diversity and Inclusion (EDI). Recommendations for change identified in the workshops were based on three topics: institutions, staff, and students.

**Conclusion:**

Our findings confirm conclusions from other institutions that staff education is urgently needed to create and maintain equitable, inclusive environments in osteopathic educational institutions in the UK to support all students, particularly those from underrepresented groups. Institutional EDI processes and policies also need to be clarified or modified to ensure their usefulness, accessibility, and implementation.

**Supplementary Information:**

The online version contains supplementary material available at 10.1186/s12909-024-05404-3.

## Background

Social injustices affecting people from minority groups have been highlighted in recent worldwide initiatives such as the ‘Black Lives Matter’ [1] and ‘Me Too’ [2] movements and investigations have identified institutional racism, sexism and homophobia in the police, other public services, and business organisations [3–6]. Limited demographic diversity and evidence of discrimination against minority groups have been reported in higher education in the United Kingdom (UK) [7] and in healthcare services including medicine, psychiatry, and physiotherapy [8–10]. Data from higher education institutions suggest there is an urgent need to improve recruitment, educational experiences, and attainment for students from minority groups [11–13].

The terms ‘minority’ or ‘under-represented’ are often used interchangeably to describe groups of people identified by specific demographic or cultural characteristics. In this paper, the term ‘under-represented’ is used to emphasise that experiences of inequity are typically created and maintained by social constructs such as ‘othering’: the process of identifying people as different from oneself or the mainstream culture, often associated with negative beliefs and expectations [14]. Social constructs can provide both unearned advantage (‘privilege’) and disadvantage (‘oppression’) [15]. Characteristics used to identify others can include skin colour, ethnicity, religion, gender identity, sexual identity, ability, size, socioeconomic status, history of trauma, addiction, and family environment [15].

People from under-represented groups (UrGs) have historically been less likely to access higher education [16], although the number of BAME, LGBTQIA + and disabled students is gradually increasing in England [17, 18]. Enrolled students from these groups are reported to experience more negative experiences during training and more limited later career opportunities afterwards [19, 20]. The General Medical Council (GMC) recently set new targets to improve access and outcomes for students from UrGs [21] as lack of diversity and limited cultural awareness among practitioners from different healthcare professions also impacts the quality and outcomes of healthcare for patients from UrGs [12, 22, 23]. The Council of Deans recently published a report on how to build an inclusive environment which highlights issues that affect students from minority ethnic groups in Allied Health Professions [24].

Patients from UrG experience substantial health disparities in the UK and across the globe due to structural and interpersonal discrimination [25, 26]. Developing cultural humility in clinicians is seen as key to bridging the gap of interpersonal discrimination. Cultural competence was once considered as an adequate way to provide an inclusive environment. It is defined as “a set of congruent behaviours, attitudes and policies that come together in a system, agency or among professionals that enable that system, agency or professions to work effectively in cross-cultural situations” ([27] p. iv). The concept shifted to cultural humility, defined as “the ability to maintain an interpersonal stance that is other-oriented (or open to the other) in relation to aspects of cultural identity that are most important to the client” ([28] p. 354).

Osteopathy is a form of manual therapy which is now recognised as one of 14 Allied Healthcare Professions in England [29]. In the UK, there are currently seven osteopathic education providers (OEPs) and approximately 5,300 qualified osteopaths. Training is typically over four or five years in the form of Bachelor’s or Integrated Masters awards and practitioners then register with the statutory regulator, the General Osteopathic Council (GOsC), and are required to comply with professional standards of practice [30].

There is little known about discrimination, bullying and harassment in osteopathy education as highlighted in a recent systematic review [31]. Therefore, the current research project aimed to assess osteopathic students’ awareness of cultural diversity and beliefs about patients from UrGs and their preparedness in managing them; to explore the educational experiences of students with UrG backgrounds during training and their opinions on changes to support better levels of recruitment and achievement. Finally, the research was disseminated to stakeholders in workshops with the overall aim of developing recommendations for action and creating change.

## Methods

### Design

To meet the multiple aims, a mixed methods approach was implemented and included the following stages; a survey of students attending all seven OEPs in the UK; focus groups with UrG students; and a workshop forum to explore the findings with diverse stakeholders. This design was based on a transformative action research paradigm with students participating as collaborators (Mertens 2007; 2010), informed by previous research into EDI, cultural competence and cultural humility in healthcare education, outlined below. The research complies with the Good Reporting of A Mixed Methods Study (GRAMMS) guidance [32] (see supplementary material 1– GRAMMS reporting).

**Fig. 1:**
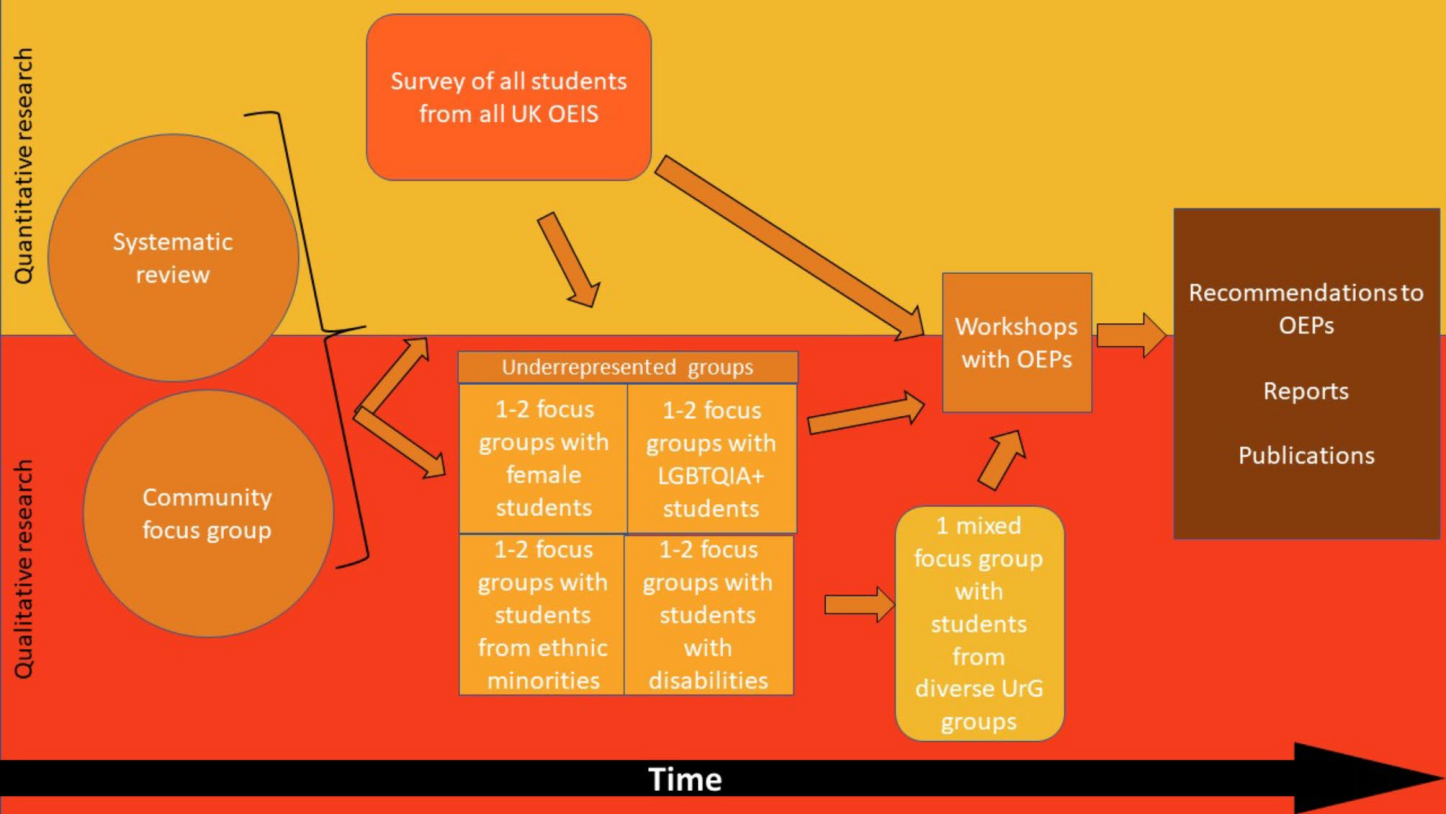
Study design

Figure 1 below details the mixed method stages with the quantitative data collection (top half of figure), qualitative data collection (bottom half), and mixed methods stages (middle). The stages are represented chronologically, starting on the left.

### Methodology

This research project sits within a transformative paradigm that places central importance on studying the lives and experiences of marginalised groups and is appropriate for addressing inequality and injustice in society [33]. An explanatory sequential mixed methods design (survey followed by focus groups) was implemented to gain insight [34] and community members were involved in initial discussions about operationalising the research focus. Transformative research has power issues and inequalities at its core and a political agenda that aims to change the experiences of the participants and institutions involved [35]. The study was approved by the University College of Osteopathy Research Ethics Committee.

### Community engagement

Two community engagement meetings with students from underrepresented groups were established prior to the project to ensure it was designed ‘with’ students rather than ‘to’, ‘about’ or ‘for’ them. Based on principles by [36], these community engagement meetings co-created the study design, modified the research questionnaire and recruitment approaches.

### Quantitative stage

A survey of all students currently enrolled on an osteopathic course in the UK was chosen to explore the research objectives. All students enrolled at the seven OEPs in the UK (excluding postgraduate and CPD courses) were eligible to take part in the anonymous online survey on Qualtrics©. Invitations, study information and accessible links were disseminated via OEP contacts who sent it to their student body between 7th and 31st March 2022. Two reminders were sent.

### Survey instrument

The Multidimensional Cultural Humility Scale (MCHS) was selected for this project as there is good evidence of convergent and discriminant validity and internal reliability [37]. The MCHS has five dimensions, contains 15 items with a 6-point Likert scale from ‘strongly disagree’ to ‘strongly agree’ where higher scores represent greater levels of cultural humility. The MCHS was used to understand to understand awareness of cultural sensitivity in the environment in which UrG students were learning. This project was not about clinical services. Modifications to the MCHS were necessary to contextualise it for osteopathy students, so a factor analysis was conducted to assess the validity of the adapted version. Following the community engagement meetings, a 7th category was added: ‘This has never crossed my mind’ to assess whether students were comfortable, confident or aware of particular issues (see supplementary material 2 for the adapted versions used in this study).

Questions related to demographics and personal characteristics (clinical or pre-clinical student, age, birth sex, gender, ethnicity, health and disability status, sexual orientation, and religion), and to their experience of education were included at the end of the MCHS and were analysed separately to the MCHS questionnaire.

### Qualitative stage

Focus groups were selected for this phase and represented four UrG: ethnic minority, disability, LGBTQIA + or women. Whilst women are not numerically under-represented in UK osteopathic undergraduate training, socially they are more oppressed than men, including in manual therapy training [31]. The choice of these four groups was discussed and agreed as important priorities in the community engagement meetings. For sensitive topics, homogeneous groups foster a sense of belonging and facilitate disclosure [38]. Focus groups usually comprise 6 to 8 people who meet once for approximately 90–120 min, and the usual number of groups is around 4 but depends on the complexity of the topic and heterogeneity of the samples [39].

Students from any UK OEP who identified as belonging to at least one UrG (ethnic minority, disability, LGBTQIA + or women) were eligible to participate with students from the same and/or other OEPs. Each OEP was responsible for forwarding invitations to participate to their students. For convenience, focus groups were conducted online as students from different OEPs were geographically dispersed [40]. The research team members acting as focus group facilitators identified with one or more minority groups, representing diversity and were therefore part of the data, as is good practice in transformative paradigmatic research [41]. All facilitators had previously used focus group methods, participated in training, or were used to managing student group discussions. Teams© created automatic initial draft transcriptions to aid later transcription if participants talked simultaneously [39]. Final transcripts only included pseudonyms, as is common in qualitative research [42]. Focus groups sessions ran for approximately 90 min. Students who had participated in one of the four initial groups were invited to join one final mixed group to discuss the previous findings, and students who participated in at least one group were invited to take part in the workshop forum.

### Dissemination forum and discussion workshops

An interactive face-to-face workshop-based forum was held on 06/04/2023 to disseminate the survey and focus group results, discuss their implications, and develop recommendations for action. Key stakeholders invited to attend free of charge included UK OEPs, the General Osteopathic Council, the Institute of Osteopathy, the Osteopathic Foundation, and other healthcare profession organisations, NHS representatives, and Health Education England. Approximately 70 people attended the event. Three interactive workshops focusing on specific aspects of EDI (students, staff and institutional governance), with different methods to promote open discussion, explored responses about ways to develop a more supportive educational environment and inclusive curriculum.

## Mixed methods analysis

### Quantitative stage

To assess whether the 5-factor model of the MCHS remained valid following changes made to the scale, a confirmatory factor analysis was carried out using using R (version 4.3.2) [43]and the R lavaan package (version 0.6–16) [44]. Missing MCHS data was imputed using multivariate imputation by chained equations [45]. MCHS data was checked for normality using QQ plots and the Henze-Zirkler test.

A sum of all MCHS items (reverse coded as appropriate) was calculated as an overall measure of cultural humility. Linear regression was carried out to determine which demographic factors influenced this total score. Additionally, a Welch Two Sample t-test [46] was carried out to determine if MCHS total score differed between clinical and pre-clinical students. Chi-squared tests, with p-values estimated by Monte-Carlo simulation, were used to test for associations between students’ report of having been treated differently one the one hand, and demographic factors on the other. Descriptive statistics were used to report survey results.

### Qualitative stage

Focus group data analysis was conducted within a reflexive thematic analysis framework [47], which aligns with a transformative paradigm (Creswell 2014). Data was co-created by participants and facilitators, and themes were co-created with analysts through their thoughtful engagement with the data [47]. After conducting one focus group with each UrG (*n* = 4), early analysis was conducted. Another 4 focus groups with different students were conducted to analyse how these participants’ experiences resonated with the initial findings. The last focus groups ran with students from mixed UrGs to discuss the findings, conduct a meta-synthesis, and prioritise what actions students thought OEPs should prioritise.

Three interactive workshops were run to explore the resonance and implications of the quantitative and qualitative findings to date. Each workshop focused on either student, staff or institutional EDI issues, although there was inevitably some overlap, and each workshop ran three times to enable participants to contribute fully. Small groups of mixed stakeholders worked took part in varied activities to discuss the study’s findings and their ideas were recorded on post-it notes, flipcharts or noted by facilitators during plenary discussions. After the workshop, written comments were collated by the facilitators (YF, HA, SV) and categorised into themes by members of the research team (JDR, HA), using frequency analysis (where data was available) to identify strong and recurring recommendations for change.

The data from the quantitative and qualitative phases were analysed separately, but then were considered together both in the forum workshops and within the research team. When considering the quantitative and qualitative datasets together, the research team operated within the methodological spirit of pragmatism, whereby both data sets were integrated in such a way that a useful insight to the research provided useful insights to participants’ experiences and generate knowledge with social utility [48]. In practice, this meant that survey results were presented to focus group participants to stimulate reflection and discussion and explore how the results compared with their personal experiences. Finally, the workshops provided an additional method to explore, situate and integrate the synthesised qualitative and quantitative data sets to support development of the final thematic model.

## Results

### Quantitative results

Two hundred and two participants filled in the survey, of which 117 (58%) were complete. The response rate was 20% (Table 1). Responses per OEP ranged between 6 and 68 (Table 2– OEP Responses).

**Table 1:** Response and completion rates

Completion	Frequency	Percentage
Incomplete	85	42%
Complete	117	58%
Total	202	100%
Total population	Ca 1000	20% response rate

**Table 2:** Response by osteopathic education provider

	Number of responses per OEP (*n* = 202)
A	10 (5%)
B	25 (12%)
C	25 (12%)
D	39 (19%)
E	6 (3%)
F	8 (4%)
G	21 (10%)
H	68 (34%)

Seventy percent of the respondents provided demographic information (*n* = 142). Participants were mostly white (*n* = 95), female (*n* = 74), without a disability (*n* = 106), heterosexual (*n* = 89), and identifying with no religion (*n* = 69) (see Table 3– respondent demographics).

**Table 3:** Respondent demographics

Category	n= (total 142)	%
**Sex at birth**		
Female	77	54.2
Male	40	28.2
Intersex	0	0
Prefer not to say	2	1.4
Not answered	23	16.2
**Gender**		
Woman	74	52.1
Man	41	28.9
Non-binary / Gender-variant / Non-conforming	1	0.7
Transgender man	0	0
Transgender woman	0	0
Not listed	1	0.7
Prefer not to say	2	1.4
Not answered	23	16.2
**Ethnicity**		
White	95	66.9
Mixed or Multiple ethnic groups	7	4.9
Asian or Asian British	6	4.3
Black, African, Caribbean or Black British	5	3.5
Other ethnic groups	1	0.7
Prefer not to say	5	3.5
No response	23	16.2
**Disability**		
No	106	74.7
Yes (details below, several answers possible per participant)	11	7.7
* Mental health conditions and illnesses*	*5*	*3.5*
* Developmental impairment, such as autistic spectrum disorders (ASD), dyslexia and dyspraxia*	*4*	*2.8*
* Impairments with fluctuating or recurring effects (such as rheumatoid arthritis, myalgic encephalitis (ME), chronic fatigue syndrome (CFS), fibromyalgia, depression and epilepsy)*	*2*	*1.4*
* Sensory impairments (such as those affecting sight or hearing)*	*1*	*0.7*
* Progressive impairment (such as motor neurone disease, muscular dystrophy, and forms of dementia)*	*1*	*0.7*
* Auto-immune conditions (such as systemic lupus erythematosis)*	*1*	*0.7*
* Learning disabilities*	*1*	*0.7*
* Impairment produced by injury to the body, including to the brain*	*1*	*0.7*
Prefer not to say	2	1.4
No response	23	16.2
**Sexual orientation**		
Heterosexual	89	62.7
Bisexual / Pansexual	11	7.7
Gay / Lesbian	10	7.0
Other	1	0.7
Prefer not to say	7	5
No response	24	16.9
**Religion**		
No religion	69	48.6
Christian	23	16.2
Spiritual	13	9.2
Other religions	3	2.1
Buddhist	2	1.4
Muslim	2	1.4
Jewish	1	0.7
Sikh	1	0.7
Hindu	0	0
No response	28	19.7

Most participants identified to some extent with an UrG (*n* = 62, 53%). Of all the students who responded (53% self-identifying as UrG to some extent, and 47% who did not identify as UrG), 67.8% (*n* = 80) reported that they had not been treated differently because of their cultural background or identity. Those who had been treated differently (*n* = 19; 16%) stated that it happened at least a few times per year (*n* = 15, 79%) (supplementary material 3, table a– underrepresented groups treatment). Of the 28 who reported having been treated differently, 18 reported whether they had complained: 15 had not complained (6 open-ended responses: not significant enough (*n* = 2), unlikely to lead to change (*n* = 2), fear of being identified (*n* = 1), happened once and felt that mistakes happen (*n* = 1)). Six of the 15 who did not complain did not know how or to whom to complain.

Associations between demographic characteristics and UrG self-identification found that ethnicity (merging all categories excluding White), Disability and Sexual Orientation (merging all categories excluding heterosexual) were significantly associated with identifying as belonging to an UrG group (Supplementary material 3, Table b - UrG identification vs. demographic group).

No significant associations were found between demographic characteristics and reports of being treated differently (Supplementary material 3, table c - treated differently vs. demographic group).

Of the 19 participants who reported having been treated differently because of their culture or identity, 79% (*n* = 15) did not report it to their OEP, 15.8% (*n* = 3) did, and 5.2% (*n* = 1) did not answer.

It was not possible to confirm or deny the adequacy of the 5-factor model proposed by Gonzales et al. [37] (Supplementary material 4), so our analysis was based on their 5-factor model (see Table 4– MCHS results). Regarding the MCHS total score, no differences were found between clinical and preclinical students (Welch’s t = -0.194, df = 79.3, *p* = 0.847). A weak correlation between MCHS total score and importance to individual was found (Spearman’s rho(114) = 0.27, *p* = 0.003), and a weak relationship between self-rating of skills and MCHS total score (rho(114) = 0.26, *p* = 0.005). There was no apparent relationship between MCHS total score and participants’ perception of support in the clinical environment for exploring patients’ backgrounds and experiences (rho(106) = 0.097, *p* = 0.3). No scores on these three questions differed significantly between clinical and preclinical students.

**Table 4:** MCHS results

MCHS dimension	Mean	SD	Median	IQR
Openness	14.967	2.289	15	2.00
Self-awareness	14.128	3.15	15	3.75
Person-centred	11.364	3.49	12	5.00
Therapeutic interactions	10.861	2.75	11	4.00
Reflective practice	16.233	1.606	17	2.25

### Qualitative results

**Fig. 2:**
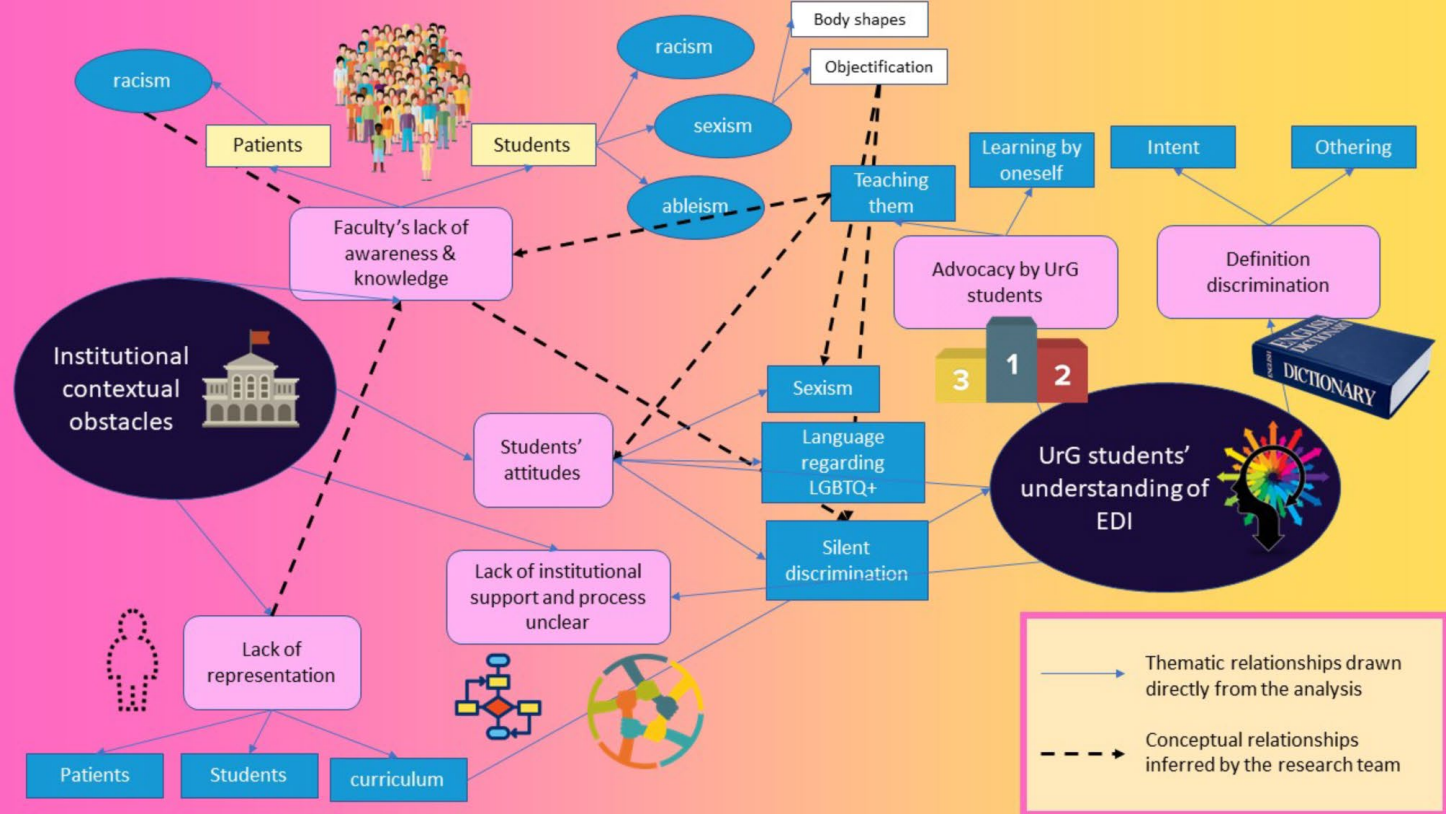
Model based on focus groups’ themes and sub-themes

Seven groups were conducted, each were facilitated by two members of the research team (from AMM, HA, JDR, SV, YF). Data from the first six focus groups were organised into two themes which provide descriptive insights of participants’ reflections on the quantitative findings and how these results related to and resonated with their own experiences. The two primary themes were named institutional contextual obstacles (with 4 sub-themes) and UrG students’ conceptual understanding of EDI (with 3 sub-themes). The themes and sub-themes were modelled and presented to the final focus group to facilitate reflective discussions, see Fig. 2.

#### Theme 1: Institutional contextual obstacles

The first sub-theme, *Faculty’s lack of awareness & knowledge*, was a commonly reported barrier.

*I think there’s a lot of talk of self-reflection, at least at the OEP, and it doesn’t to me feel like all of our teachers practise that.*

I’ve had more problems with staff understanding than student understanding”. (Talking about their disability)

There was no awareness, you know, of that within the class or from the tutors, in those circumstances (managing an LGBTQ + patient), what do we do, what language do we use, (…) when it was raised the tutor was sort of like, actually, I don’t have an answer, I’m not sure.

Racist, sexist and ableist comments made by staff negatively affected the way students interacted with patients in the OEPs clinics, and with other students, particularly in practical classes.

I was doing a neck and then teacher wants me to talk when I’m doing it and I say, because / when I’m doing it, I can’t talk and he made a comment, as a woman, you should talk and you should do it, you should multi-task and at that time I couldn’t say anything because I [was] already panicking and I’m doing this thing. I couldn’t say anything.

[A male tutor] put [a female tutor acting as a model] side-lying and [he] was going to crack her back but then when he pulled her shirt up her scrubs pants were like mid-way / quite / kind of showing her underwear (…) When we told him that he should pull her scrubs up, he made the thing super uncomfortable.

When they make an attack, as a joke, and people laugh, that’s positive behaviour, they’re going to make the joke again because it’s funny, so I don’t know if they can understand that it’s actually a knife that you’re throwing at someone and not just a joke.

The second sub-theme related to a lack of support from institutions for students from UrG, and a lack of clarity of processes available to them to complain about discriminatory behaviours against them.

When I was sort of going through the process of applying for the disabled students’ allowance, which I didn’t even know that I was / its existence to be honest, (…) I had to get the OEP to fill out a form and rubber stamp it and it seemed to get lost in this abyss of I don’t know where it went. (…) but there was a lot of chasing up to do [laughs] and even getting the form signed again, because I have to reapply every year, was a bit of a faff.

Participants who reported discrimination, were lacking certainty that reported these instances would lead to change.

Particularly when it’s a comment like that that’s made and it almost leaves you like gobsmacked and you’re like well what do I say to that, how do I go about telling someone about that?

The third sub-theme was *Student attitudes* e.g., peers making sexist comments and using negative language about UrGs.

People have said things, especially kind of bisexual tropes and things like that about you know being greedy and I know it’s / (…) people think oh that’s funny (…) it just makes you feel like you are going inward kind of thing.

I was practicing thoracic HVT with (…) some first years [students] and I started doing thoracic HVT and one of the first years asked me to do it on him, so I was like, okay, umm, I explained to him you know everything, asked for his consent and stuff, but because he was like a funny guy, he was talking all the time, I was like, okay, can you just sit down for me to do the technique and I told him my nationality before that and then he goes like oh that’s how I know you are Brazilian, your attitude, you probably go on top. I’m just like what? You know / yes, I didn’t even know what to say at this time, because I was just / I just told him, look, I’m not doing the technique, I thought, goodbye.

Participants reported instances where students from privileged backgrounds remained silent when facing discriminatory comments from educational faculty; a factor that perpetuated a non-inclusive culture, as people who used discriminatory or ‘othering’ language were not challenged to reflect on their attitudes and behaviour. In contrast, participants from UrGs felt a sense of duty to raise concerns:

I don’t create problems and stuff, but if there is something if I see it not going right, I like to raise my voice as much as I can and I try to make changes.

The fourth sub-theme was *Lack of representation* in the student body, patient population and the curriculum.

Everything that we get taught is 99% on like a male sex anatomy. Like I remember when I was learning how to do all the like umm cardiac testing and respiratory we were taught by a male teacher on a male body and then when it came to like a female and like you have boobs and they’re like, oh, you can’t do this bit at the front, or you have to be more careful, but then there was no example of how.

I think I felt surprised when coming into the / into osteopathy how less diverse (in student demographics? ) it is than my previous position.

I feel quite diverse but people that we see in clinic are mainly Caucasian, so I also think there’s something about the outreach of osteopathy into different cultural communities, for example, most of my family, though we’ve all been brought up here, nobody would use an osteopath (…).

When we learn about physiology and pathologies, I feel like there’s now a real effort to talk about say like black people, which is fantastic, but then you know what about Asian (…).

#### Theme 2: Underrepresented students’ understanding of EDI

The first sub-theme related to the *definition of discrimination* and echoed findings from community engagement discussions. Students distinguished between ‘othering’ and ‘intent’. Participants perceived discrimination only when actions had an intent to discriminate against individuals or communities, rather than actions that led to people or groups being treated differently regardless of intent. During the focus groups, participants reported equal treatment, but data analysis suggests instances of discrimination.

No, only in so much as, you know, the reasonable adjustments aspect, but then I’ll ask for that, but besides that, I haven’t / I haven’t had any different treatment.

I’ve definitely been treated differently as a woman and / but I’ve witnessed the / in my class Asian women being treated differently, but the Asian men not so much so.

The second sub-theme related to the advocacy of UrG students as role models for their peers. Students used their own experience of belonging to an UrG as personal knowledge to help inform their peers about what it is like to *be* a person from wider UrG communities. This helped to fill gaps in the EDI training or make up for a lack of training received by educators. UrG students acted as advocates to prevent wrong messages, jokes being shared, e.g.,

I think it’s / not just from my disability, but yes, from / for all other students I think when they / things come up, sometimes quite surprising things actually, it’s usually / yes, pretty interesting and helpful for all of us.

We use it [disability] sometimes in class as part of like chronic pain, as part of that kind of presentation and things like that because I have an understanding of it, whereas instead of just pulling stories out of thin air.

The third related sub-theme was that students from UrGs appeared to have a better understanding of EDI than their peers and faculty members. Students’ advocacy role included training and supporting their peers in how they should manage situations when facing patients with specific conditions, e.g. type 1 diabetes, and offered a useful insight which would be valued by patient.

I mean do they have to? Should they? I think you know, like I’ve said, the only reason I do [disclose] it is because you know I wouldn’t want to put anybody else in a tricky position if I was to, you know, have like a hypo in class or anything like that, which you know, I may do one day.

This created an environment where students from UrGs not only had to teach other students and faculty, but also had to learn on their own, as they were not able to gain knowledge from staff on topics related to UrG, and then had to teach what they learned to their peers and faculty members.

But we don’t get taught about how to deal with somebody that’s transgender or anything like that. It’s like well you’ll have to you know just find out about that yourself.

I don’t have that much of an understanding of the difference that ethnicity has on sort of different diseases and different morphologies and things like that, so it’s something (…) I’d love to learn more about.

The final mixed focus group was used to explore whether the above findings represented the experiences of these participants, and to generate suggestions for OEP action to become more inclusive. Goals thought to be quickly achievable and likely to lead to sustained change was providing urgent training for staff, and then students, to improve awareness and knowledge, and to break the issue of the cycle of unaware students becoming unaware teachers.

Lack of diversity ‘breeds’ a lack of diversity.

A lot of the main institutional barriers is the university’s lack of knowledge and the best way to deal with that is directly linked to how the under-represented students can like just you know break this barrier by teaching others and also by getting contact with the university.

Active bystander training was recommended to promote collective responsibility in challenging bias and negative views. Other suggestions included providing support for students from UrGs, countering negative views amongst peers and faculty, employing active strategies to promote patient diversity, being more equitable in services offered, and ensuring training was implemented. The final recommendation was to increase representativeness in the curriculum, as a way of training staff and students through regular exposure to up-to-date information regarding UrGs.

if the institutions were to be more aware [of EDI] and have [EDI training]…., I don’t know what training’s mandatory training’s given, but it would seem like potentially a lot of it [othering] could potentially be stopped. It just seems because you’ve got the lack of representation to faculty, race in faculty, they all sort of interlink with the other parts.

Participants felt that more and better training was needed for staff on EDI issues; a potential barrier to implementation was time, but short courses were expected to be effective.

every job I’ve ever done, either private sector, public sector, there is mandatory training and EDI’s, (…) human trafficking, (…) blackmail. (…) But I think we’re only talking like a half an hour.

### Workshop results

Comments from nine workshop sessions (three each on student, staff and institutional EDI issues) were combined using frequency analysis to identify key themes and recommendations for change (Table 5). The strongest theme addressed stakeholders’ opinions about staff issues (96 comments in total), with recommendations about the need to improve staff attitudes [36], increase their awareness of students’ needs [15], and enhance communication skills [26]. The second main theme was student support [49], including the need to explore barriers to change [26] and improve access to support services [14]. Two other themes focused on the need to clarify and improve institutional EDI policies and processes [26] and ways to improve representation and diversity among student osteopaths, OEP staff and patients seeking osteopathic treatment [25] (also see Supplementary Material 5).

**Table 5:** Combined summary of workshop feedback

Workshop feedback about EDI issues (+ frequency counts)	Recommendations for positive action
**Theme 1: Lack of EDI awareness among staff (96)** Examples of poor staff attitude and behaviour towards URGs (36) Inappropriate communication between staff and students (26) Little awareness of URG students’ learning and support needs (15) Inconsistency/lack of EDI in clinical and academic education (13) Unclear policies/processes for EDI monitoring and governance (6)	Provide EDI and communication skills training. Share information about EDI values, policies and best practices. Address EDI concerns and communicate actions effectively. Collect data on student demographics, EDI attitudes/issues. Create culturally diverse tutorials, patient cases, presentations. Embed EDI in staff recruitment, induction and progress reports.
**Theme 2: Inconsistent or ineffective student support** (51) Institutional barriers and resistance to change (26) Unclear access to relevant support (14) Limited sharing of information and limited available support (9) Structural/institutional barriers to diversity and inclusion (2)	Integrate expectations about EDI in student support at all levels. Provide rolling programme of ‘Active Bystander’ training for all. Develop and deliver an effective Personal Tutor system. Communicate the actions taken about EDI issues identified. Collect data about structural, environmental, clinical challenges.
**Theme 3: Lack of clarity in institutional polices and processes** (26) Lack of clarity about complaints policies and processes (9) Lack of clarity about professional values and governance (7) Lack of clarity about assessment and feedback processes (5) Lack of clarity about acceptable language and behaviour (5)	Make EDI policies and processes accessible and visible. Clarify the possible/actual outcomes of complaints processes. Review and share information about institutional values. Clarify policies for complaints, whistleblowing, and impact. Review policies about acceptable language and behaviour.
**Theme 4: Poor representation for minority groups** (25) Limited diversity/representation in student assessments (17) Limited understanding of patients from UrGs (7) Limited diversity in OEP staff (1)	Make institutional marketing images more diverse and inclusive. Create collaborative feedback methods, esp. with UrG students. Develop the clinical curriculum to reflect social diversity. Review implicit bias in staff and student recruitment.

**Fig. 3:**
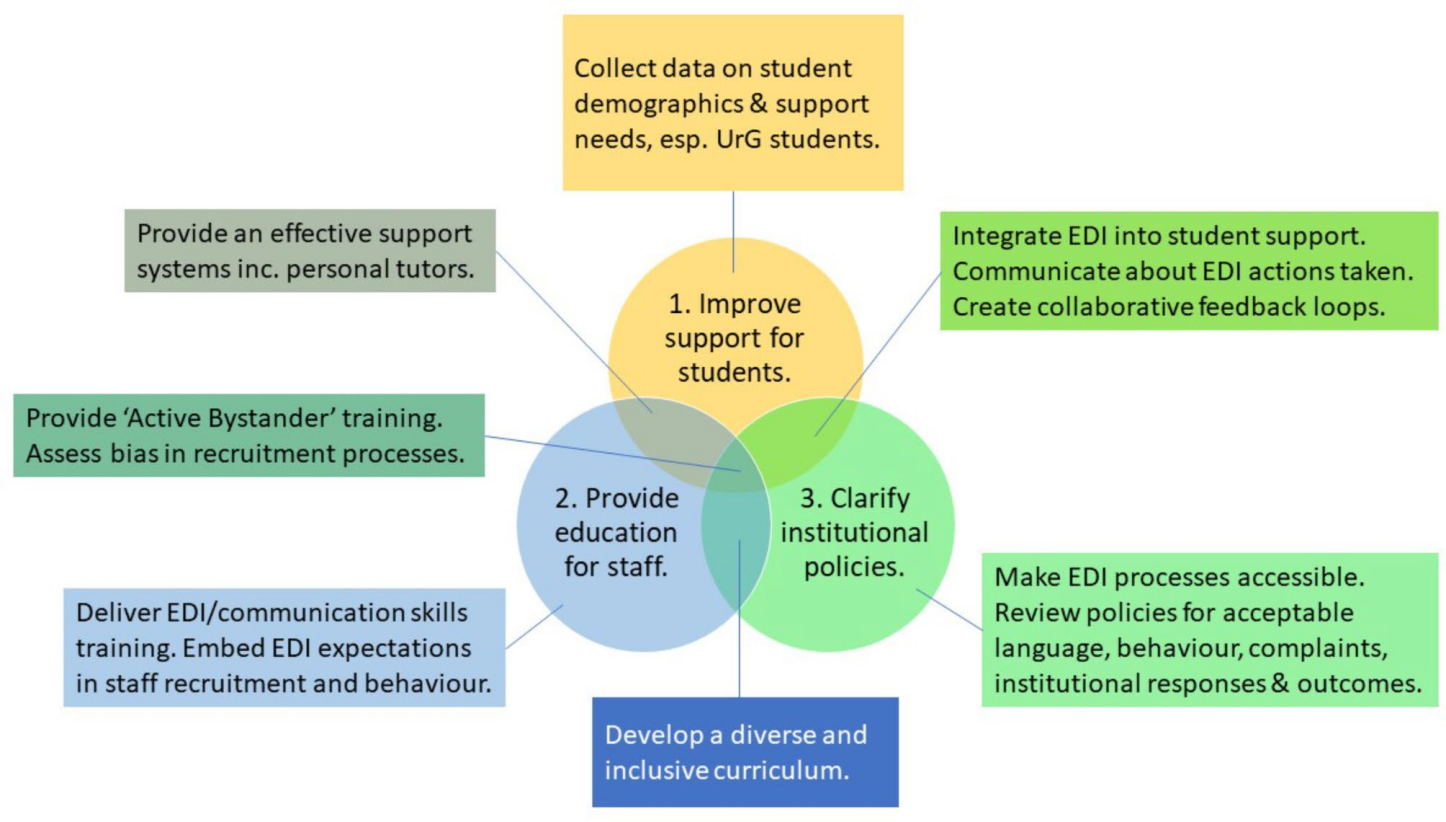
Workshop themes

Overlapping themes were organised in Fig. 3 in relation to the groups involved in the recommended actions.

## Discussion

The aims of this innovative mixed methods study were to survey student osteopaths’ levels of cultural humility to assess levels of awareness in the current educational environment and as a proxy for preparedness to work with patients from diverse backgrounds. It also explored the educational experiences of UrG students with the aim of improving equity, diversity and inclusivity (EDI) and sense of belonging in Osteopathic Educational Providers (OEPs). The survey response rate was 20%, but data was collected from 202 students from seven OEIs. 62 students identified with at least one UrG and 19 reported that they had been treated differently but 15 had not reported it.

Qualitative data from focus groups with students from the four selected UrGs suggested the main challenges faced were staff attitudes and lack of awareness; limited student support; and lack of representation in the curriculum and in institutional processes. These themes were explored and refined in interactive workshops, which generated recommendations to improve staff education, support students, and develop effective institutional policies. The implications of these findings are discussed below.

### Educating staff

Cultural humility is a lifelong commitment to developing awareness to disparities experienced by people from diverse cultural groups, reflecting and being open to learning [49–52]. This model encourages practitioners to collaborate with patients, and educators to collaborate with students, to find solutions to discrimination and inequality based on their lived experiences and priorities [53]. Qualitative findings from the focus groups and workshops in this study indicated that experiences of ‘othering’ and discrimination were often associated with lack of cultural humility, self-awareness, ignorance, or overtly negative attitudes, mainly among staff. (Focus group theme 1: “*I don’t know if they can understand that it’s actually a knife that you’re throwing at someone and not just a joke*”).

There is limited evidence exploring the impact of cultural humility training with healthcare professional educators. Bakaa et al. [54] surveyed cultural competence in a sample of 3,000 chiropractors and reported similar findings which suggested that gaps between knowledge and self-reported behaviour required further research to clarify barriers and guide future training. Flateland et al. [55] concluded that inclusivity could be increased through mandatory diversity training which emphasised individual learning needs for students from all backgrounds and was supported by mentoring from personal academic tutors and a buddy system for UrG students.

A focus group study by Shapiro et al. [56] suggests that training increased awareness among third year medical students (first year of clinical training) but was less helpful in developing specific management skills. In contrast, another study found that, medical students tended to minimise the importance of self-awareness or the need to reflect on, and confront, personal biases [50]. Despite uncertainty about the impact of training, there is consensus that lack of training is also problematic. Whether based on concepts of cultural awareness, competence and humility [51] it is important that the sceptical perception that training is trying to be ‘politically correct’ is transformed into a way of rehumanising healthcare education [56]. Education in EDI and inclusive communication skills was strongly recommended by the participants in this study, but the challenges cited above suggest that ongoing monitoring would be needed to explore its’ impact on staff and students (Focus group theme 2: “*I don’t know what mandatory [EDI] training’s given, but it would seem like potentially a lot of it [othering] could potentially be stopped*”).

### Supporting students

Inequalities in healthcare education are well documented [11, 12, 16, 18]. Physiotherapy students from black, Asian and minority ethnic (BAME) backgrounds received lower marks in observed assessments compared to white students, with gaps in attainment also recorded for people with disabilities and students with non-traditional entry routes [10]. Overseas students, especially those who do not speak English as a first language, report isolation, loneliness, and lack of support, which is increased by intersectionality including race and gender [9, 57]). In the survey, 15 students who felt they had been treated differently because of UrG characteristics did not report their difficulties, sometimes because they were unclear about whether an incident would count as discrimination or whether reporting a problem would have negative consequences (Workshop theme 3: “*Need to clarify what language/behaviour (e.g., ‘banter’) is acceptable*”).

Barriers to reporting misconduct include fear of not being believed, fear of repercussions and lack of confidence that complaints will be taken seriously [58]. Focus group and workshop comments suggested that students felt concerns were ignored, whether reported by individuals or year group representatives. The institution was rarely seen to take action to address the problems identified and there were concerns about consequences for people who spoke out. In contrast, some participants felt that whistleblowers should be valued and that incidents of discrimination could be reduced by encouraging more people to speak up (Workshop theme 4: “*Value all experiences and validate the ‘disruptor’ voice*”).

Research suggests that some of the factors that hinder the delivery of effective student support include limited disclosure of individual difficulties, especially for ‘invisible’ disabilities [18], the complex challenges faced by students with intersectional backgrounds [59, 60], and lack of staff awareness, as discussed above [20, 61]. Inconsistent institutional support practices also reinforce students’ disabled status and limit participation, rather than optimising their abilities and resilience [61], so there is a need to develop clear, robust systems to support students from UrGs, such as Active Bystander training (Workshop theme 2).

### Improving institutional policies and processes

The practical processes used to support students and manage staff are grounded in an institution’s values and policies. Training inequalities are known to be a concern in medical and allied health professions and all HEIs in the UK have a responsibility to overcome the challenges of inaction in the face of discrimination. The General Medical Council has recently set new targets to eradicate disadvantage and discrimination in medical education and training [21]. Equality, diversity and inclusion pose challenges for small specialist universities, as noted in the ‘Changing the Culture’ (2016) framework, developed by Universities UK and GuildHE [62]. OEPs are expected to cultivate and maintain a culture of inclusion between staff, students and patients, train staff in EDI and ensure that staff are involved in the development of EDI policies [63]. This is reflected in the Quality Assurance Agency for Higher Education Subject Benchmark Statement for Osteopathy [64]: expectations and guidance on how OEPs can promote an EDI culture are provided. Participants in this study reported concerns about institutional knowledge (Focus group theme 2) and lack of clarity about how to access and use existing EDI policies (Focus group theme 1: “*How do I go about telling someone about that?*”).

In recent decades, access and participation from minority groups to higher education in the UK has been a core focus and entry rates for non-white students have increased: in 2019 they were higher for all ethnic groups compared with rates in 2006 and the entry rates increased in 2019 compared with 2018 [65]. There is limited information about experiences of inequalities reported by UrG students in osteopathic education or discrepancies in levels of attainment. A systematic review by MacMillan et al. [31] analysed discrimination, bullying and harassment in manual therapy education. They reported that there was evidence of widespread discrimination, harassment and bullying within manual therapy education; and there was a clear need for further research to focus upon the intersection of the characteristics identified as being linked to these experiences. Unfortunately, no osteopathic studies were found, although findings from physiotherapy and chiropractic education are likely to be transferable. Practising osteopaths from UrGs are also reported to be dissatisfied with lack of diversity within the profession and concerns have been raised about a lack of cultural competence training in OEPs [66].

Norris et al. [61] recommended that healthcare education institutions need to provide consistent and accessible information to help students find appropriate support and education to increase staff awareness about how individual experiences of disability affect learning. Complex EDI issues require university-wide approaches and AdvanceHE’s UK Equality Charter team proposes an ‘holistic approach’ [62]. Further research is needed to identify actions which would enhance educational experiences and outcomes for student osteopaths from UrGs. New data would also provide insights into the extent that osteopathic education prepares students to work with patients from UrGs and support long-term plans to enhance access and quality of patient care and attract more students from these UrGs to enhance the profession and represent more inclusively the communities they serve [31, 64].

### Limitations of the study

It is difficult to collect data from people who feel marginalised or vulnerable to discrimination, as demonstrated by low survey response rates with participants who typically have strong positive or negative views but few from the ‘silent majority’ (Shapiro et al. 2016). The MCHS is a new instrument which was adapted to osteopathy students, and due to the small sample size, it was not possible to get useful results with the confirmatory factor analysis. More research is also needed with this instrument to establish meaningful scores for dimensions of questionnaire. The response rate to this survey was low at 20% and there were fewer than 8 participants in all the focus groups. However, mixed designs enable compensation for some limitations of individual methods and data was collected from all seven UK OEPs. Two stages of qualitative analysis (focus groups and workshops) also enabled triangulation of the findings. The impact of facilitators as ‘insiders’ on data collection was not assessed and it was challenging to synthesise and weight results from the three stages.

## Conclusions

The aims of this mixed methods study were to assess awareness of cultural humility among student osteopaths in the UK and to explore educational experiences of discrimination and ‘othering’ among students from underrepresented groups. Our findings are consistent with conclusions from other studies and the suggestions for action generated in workshops with diverse stakeholders are aligned with current EDI guidelines. Our three main recommendations are that OEIs prioritise actions to clarify institutional policies and processes to ensure they are accessible and effective in maintaining an inclusive educational environment; to review the adequacy of current student support services, particularly for underrepresented groups; and to provide EDI and communications skills training for staff to increase awareness about students’ learning needs and explore attitudinal barriers to change.

### Electronic supplementary material

Below is the link to the electronic supplementary material.

Supplementary Material 1

Supplementary Material 2

Supplementary Material 3

Supplementary Material 4

Supplementary Material 5

Supplementary Material 6

## Data Availability

The datasets generated and/or analysed during the current study are not publicly available due to the sensitivity of data collected and risk of identification of participants but are available from the corresponding author on reasonable request.
