# Correction: Overcoming barriers to equality, diversity, inclusivity, and sense of belonging in healthcare education: the underrepresented groups’ experiences in osteopathic training (UrGEnT) mixed methods study

**DOI:** 10.1186/s12909-024-05568-y

**Published:** 2024-06-07

**Authors:** Jerry Draper-Rodi, Hilary Abbey, John Hammond, Oliver P. Thomson, Kevin Brownhill, Andrew MacMillan, Yinka Fabusuyi, Steven Vogel

**Affiliations:** 1 University College of Osteopathy, 275 Borough High Street, London, SE1 1JE UK; 2 National Council for Osteopathic Research, 275 Borough High Street, London, SE1 1JE UK; 3 School of Allied and Public Health Professions, North Holmes Road, Canterbury, Kent, CT1 1QU UK; 4 University of Portsmouth, University House, Winston Churchill Ave, Hampshire, Portsmouth, PO1 2UP England

**Correction: BMC Medical Education (2024) 24:468** 10.1186/s12909-024-05404-3

Following publication of the original article [1], the authors identified an error in the author name Oliver P. Thomson.

The incorrect author name is: Oliver T. Thomson.

The correct author name is: Oliver P. Thomson.

The original article has been corrected.
