# Supplementary material for: Overcoming barriers to equality, diversity, inclusivity, and sense of belonging in healthcare education: the Underrepresented Groups’ Experiences in Osteopathic Training (UrGEnT) mixed methods study
Source: BMC Med Educ. 2024 Apr 26;24:468. doi: 10.1186/s12909-024-05404-3 (PMC11055260; doi:10.1186/s12909-024-05404-3)
Supplement: Supplementary file 1 — Supplementary Material 1 [file 12909_2024_5404_MOESM1_ESM.docx]

| Good Reporting of A Mixed Methods Study (GRAMMS) | Pages where information is provided in the manuscript |
| --- | --- |
| (1) Describe the justification for using a mixed methods approach to the research question | p.4 (in qual research analysis plan |
| (2) Describe the design in terms of the purpose, priority and sequence of methods | p. 2 (in Design) |
| (3) Describe each method in terms of sampling, data collection and analysis | pp. 2-4 (Quant and Qual stages sections) |
| (4) Describe where integration has occurred, how it has occurred and who has participated in it | p.4 (regarding synthesis focus group and forum) |
| (5) Describe any limitation of one method associated with the present of the other method | p. 14 (limitations of the study) |
| (6) Describe any insights gained from mixing or integrating methods | pp. 4-5 (last paragraph of methods section)  p. 12 (workshop results)  p. 14 (limitations of the study) |
