# Supplementary material for: Overcoming barriers to equality, diversity, inclusivity, and sense of belonging in healthcare education: the Underrepresented Groups’ Experiences in Osteopathic Training (UrGEnT) mixed methods study
Source: BMC Med Educ. 2024 Apr 26;24:468. doi: 10.1186/s12909-024-05404-3 (PMC11055260; doi:10.1186/s12909-024-05404-3)
Supplement: Supplementary file 2 — Supplementary Material 2 [file 12909_2024_5404_MOESM2_ESM.docx]

**Supplementary material**

*The questions on the left are from the original Multidimensional Cultural Humility Scale by (Gonzalez et al 2021) which was designed for qualified healthcare practitioners, and on the right is the adapted version used in this study with osteopathic students. PP.2-3 is the modified questionnaire for students managing patients, pp4-5 is the modified questionnaire for students not yet managing students.*

*Participants answered on 7-point Likert scale where 1 represents strongly disagree, 2 represents disagree, 3 represents slightly disagree, 4 represents slightly agree, 5 represents agree, and 6 represents strongly agree; and ‘This has never crossed my mind’ with reverse coding for negatively phrased questions. Higher scores represent greater levels of cultural humility.*

**Multidimensional Cultural Humility Scale modified** *(clinical students)*

**Items rated on a six-point Likert scale, ranging from strongly disagree to strongly agree, with an option to select “this has never crossed my mind”. Higher scores represent greater levels of cultural humility.**

**Openness**

- I am comfortable asking my patients about their cultural experience.
- I seek to learn more about my patients’ cultural identity.
- I believe that learning about my patients’ cultural background will allow me to better help my patients.

**Self-Awareness**

- I seek feedback from my clinic educators when working with diverse patients.
- I incorporate feedback I receive from colleagues and clinic educators when I am faced with problems regarding cultural interactions with patients.
- I am known by colleagues to seek advice when working with diverse patients.

**Multidimensional Cultural Humility Scale**

**Items rated on a six-point Likert scale, ranging from strongly disagree to strongly agree. Higher scores represent greater levels of cultural humility.**

**Openness**

- I am comfortable asking my clients about their cultural experience.
- I seek to learn more about my clients’ cultural background.
- I believe that learning about my clients’ cultural background will allow me to better help my clients.

**Self-Awareness**

- I seek feedback from my supervisors when working with diverse clients.
- I incorporate feedback I receive from colleagues and supervisors when I am faced with problems regarding cultural interactions with clients.
- I am known by colleagues to seek consultation when working with diverse clients.

**MCHS modified** *(clinical students) –* cont’d

**Person-centred**

- I ask my patients about their cultural perspective on topics discussed during treatment sessions.
- I ask my patients to describe their presenting problem based on their cultural background.
- I ask my patients how they cope with problems in their culture.

**Therapeutic Interactions**

- I wait for others to ask about my biases for me to discuss them. (Reverse coded)
- I do not necessarily need to resolve cultural conflicts with my patients during treatment sessions. (Reverse coded)
- I believe the resolution of cultural conflict in treatment sessions is the patient’s responsibility. (Reverse coded)

**Reflective practice**

- I enjoy learning from my weaknesses.
- I value feedback that improves my clinical skills.
- I evaluate my biases.
- This topic is important to me.
- I have the skills for asking patients about their backgrounds and experiences.
- The clinical environment in my institution is appropriate for to support asking about patients’ backgrounds and experiences.

**MCHS – cont’d**

**Ego-less**

- I ask my clients about their cultural perspective on topics discussed in session.
- I ask my clients to describe the problem based on their cultural background.
- I ask my clients how they cope with problems in their culture.

**Supportive Interactions**

- I wait for others to ask about my biases for me to discuss them. (Reverse coded)
- I do not necessarily need to resolve cultural conflicts with my client in counseling. (Reverse coded)
- I believe the resolution of cultural conflict in counseling is the clients ’ responsibility. (Reverse coded)

**Self-Reflection and Critique**

- I enjoy learning from my weaknesses.
- I value feedback that improves my clinical skills.
- I evaluate my biases.

**Multidimensional Cultural Humility Scale**

**Openness**

- I am comfortable asking my clients about their cultural experience.
- I seek to learn more about my clients’ cultural background.
- I believe that learning about my clients’ cultural background will allow me to better help my clients.

**Self-Awareness**

- I seek feedback from my supervisors when working with diverse clients.
- I incorporate feedback I receive from colleagues and supervisors when I am faced with problems regarding cultural interactions with clients.
- I am known by colleagues to seek consultation when working with diverse clients.

**Ego-less**

- I ask my clients about their cultural perspective on topics discussed in session.
- I ask my clients to describe the problem based on their cultural background.
- I ask my clients how they cope with problems in their culture.

**Multidimensional Cultural Humility Scale modified** *(non-clinical students)*

**Openness**

- I anticipate being comfortable asking my patients about their cultural experience.
- I anticipate seeking to learn more about my patients’ cultural background.
- I believe that learning about my patients’ cultural background will allow me to better help my patients.

**Self-Awareness**

- I anticipate seeking feedback from my clinic educators when working with diverse patients.
- I anticipate incorporating feedback I receive from colleagues and clinic educators when I am faced with problems regarding cultural interactions with patients.
- I am known by colleagues to seek advice when working with diverse colleagues.

**Person-centred**

- I anticipate asking my patients about their cultural perspective on topics discussed during treatment sessions.
- I anticipate asking my patients to describe their presenting problem based on their cultural background.
- I anticipate asking my patients how they cope with problems in their culture.

**MCHS modified** *(non-clinical students) –* cont’d

**Therapeutic Interactions**

- I wait for others to ask about my biases for me to discuss them. (Reverse coded)
- I do not anticipate needing to resolve cultural conflicts with my patients during treatment sessions. (Reverse coded)
- I believe the resolution of cultural conflict in treatment sessions is the patient’ responsibility. (Reverse coded)

**Reflective practice**

- I enjoy learning from my weaknesses.
- I value feedback that improves my clinical skills.
- I evaluate my biases.
- This topic is important to me.
- I have the skills for asking patients about their backgrounds and experiences.
- The clinical environment in my institution is appropriate for to support asking about patients’ backgrounds and experiences.

**MCHS – cont’d**

**Supportive Interactions**

- I wait for others to ask about my biases for me to discuss them. (Reverse coded)
- I do not necessarily need to resolve cultural conflicts with my client in counseling. (Reverse coded)
- I believe the resolution of cultural conflict in counseling is the clients’ responsibility. (Reverse coded)

**Self-Reflection and Critique**

- I enjoy learning from my weaknesses.
- I value feedback that improves my clinical skills.
- I evaluate my biases.
