# Supplementary material for: Overcoming barriers to equality, diversity, inclusivity, and sense of belonging in healthcare education: the Underrepresented Groups’ Experiences in Osteopathic Training (UrGEnT) mixed methods study
Source: BMC Med Educ. 2024 Apr 26;24:468. doi: 10.1186/s12909-024-05404-3 (PMC11055260; doi:10.1186/s12909-024-05404-3)
Supplement: Supplementary file 3 — Supplementary Material 3 [file 12909_2024_5404_MOESM3_ESM.docx]

Table a - underrepresented groups treatment

| Item and scale | n | % |
| --- | --- | --- |
| To what extent do you feel you belong to/you are part of an underrepresented group in osteopathic education? | | |
| Not at all | 56 | 47.5 |
| Very little | 32 | 27.1 |
| Somewhat | 21 | 17.8 |
| To a great extent | 9 | 7.6 |
| (Total) | (118) | (100) |
| Have you been treated differently based on your cultural background / identity? | | |
| No | 80 | 67.8 |
| Yes | 19 | 16.1 |
| Unsure | 19 | 16.1 |
| (Total) | (118) | (100) |
| How often have you been treated differently during training based on your cultural background / identity? | | |
| Less than once a year | 4 | 21.1 |
| A few times a year | 10 | 52.5 |
| A few times a month | 3 | 15.8 |
| At least once a week | 1 | 5.3 |
| Almost everyday | 1 | 5.3 |
| (Total) | (19) | (100) |
| Who treated you differently? | | |
| Clinic educator staff | 8 | 28.6 |
| Academic staff | 7 | 25 |
| Colleagues | 7 | 25 |
| Patients | 3 | 10.7 |
| Administration staff | 2 | 7.1 |
| Other | 1 | 3.6 |
| (Total) | (28) | (100) |

Table b – underrepresented group identification Vs. Demographic Group

| Demographic group | Chi-squared | p-value |
| --- | --- | --- |
| Bsex | 1.612 | 0.663 |
| gender | 8.196 | 0.233 |
| **ethnicity** | **14.966** | **0.003** |
| **disability** | **16.257** | **0.003** |
| **sexorient** | **11.124** | **0.009** |
| religion | 10.856 | 0.089 |

Table c – Treated differently Vs. Demographic Group:

| Demographic group | Chi-squared | p-value |
| --- | --- | --- |
| Bsex | 0.085 | 0.954 |
| gender | 4.613 | 0.279 |
| ethnicity | 4.795 | 0.077 |
| disability | 0.177 | 1.000 |
| sexorient | 2.732 | 0.268 |
| religion | 0.969 | 0.920 |

## MCHS Dimensions

### Summary

Table 1: Mean, SD, median and interquartile ranges (IQR) of MCHS dimensions

| MCHS dimension | Mean | SD | Median | IQR |
| --- | --- | --- | --- | --- |
| Open | 14.967 | 2.289 | 15 | 2.00 |
| Selfaware | 14.128 | 3.154 | 15 | 3.75 |
| Egoless | 11.364 | 3.494 | 12 | 5.00 |
| Suppinter | 10.861 | 2.754 | 11 | 4.00 |
| Selfrefl | 16.233 | 1.606 | 17 | 2.25 |

## Associations with being treated differently

### Summary

Table 2: Counts of responses for treated different levels

| Level | Count |
| --- | --- |
| Yes | 19 |
| Unsure | 19 |
| No | 80 |
| No response | 24 |

### Protected characteristics

Table 3: Chi-squared tests of association between being treated differently and other categorical variables. p values are computed by Monte Carlo simulation with 4000 replicates.

| Variable | \(\chi^2\) | p-val |
| --- | --- | --- |
| Bsex | 0.085 | 0.960 |
| gender | 4.613 | 0.292 |
| ethnicity | 4.795 | 0.078 |
| disability | 0.177 | 1.000 |
| sexorient | 2.732 | 0.264 |
| religion | 0.969 | 0.925 |

Chi-squared tests of association with being treated differently and various categorical variables are shown in table [3](#tab%253Achi2s). There are no significant associations at the 0.05 level of significance, although ethnicity is marginally significant at the 0.1 level.

### MCHS dimensions

Figure 1: Boxplots of MCHS dimensions vs treated differently

Table 4: ANOVAs: testing differences between MCHS dimensions and being treated differently

| Dimension | F | DF | p-val |  |
| --- | --- | --- | --- | --- |
| Open | 0.64 | 2 | 0.53 |  |
| Selfaware | 1.02 | 2 | 0.367 |  |
| Egoless | 0.44 | 2 | 0.644 |  |
| Suppinter | 1.41 | 2 | 0.252 |  |
| Selfrefl | 0.53 | 2 | 0.589 |  |
| *** p <= 0.001, ** p <= 0.01, * p <= 0.05 | | | | |

There were no significant associations between being treated differently and the MCHS dimensions (see table [4](#tab%253Amchstab)).

### Added Likert items

Figure 2: Boxplots of additional Likert items vs treated differently

Table 5: ANOVAs: testing differences between additional Likert items and being treated differently

| Dimension | F | DF | p-val |  |
| --- | --- | --- | --- | --- |
| newQs1 | 1.48 | 2 | 0.232 |  |
| newQs2 | 2.17 | 2 | 0.119 |  |
| newQs3 | 4.54 | 2 | 0.013 * |  |
| *** p <= 0.001, ** p <= 0.01, * p <= 0.05 | | | | |

There was a significant association between responses to the question “The clinical environment in my institution is appropriate to support asking about patients’ backgrounds and experiences” and being treated differently. Post-hoc Tukey tests showed that this association was due to a difference in those who responded ‘Unsure’ and those you responded ‘No’ (‘Unsure’ - ‘No’ = -0.949 [-1.772 -0.127, 95% C.I.]), suggesting those who were unsure whether the statement applied to them were more likely to think of themselves as being treated differently than those who did not think this statement applied to them.

### Under-represented

Table 6: Contingency table: Treated differently vs. Underrepresented (observed/expected). Expected values rounded to the nearest integer.

|  | Treated differently | | |
| --- | --- | --- | --- |
|  | No | Unsure | Yes |
| **Underrepresented** | | | |
| Not at all | 46/38 | 9/9 | 1/9 |
| Very little | 19/22 | 6/5 | 7/5 |
| Somewhat | 13/14 | 2/3 | 6/3 |
| To a great extent | 2/6 | 2/1 | 5/1 |

There is was a statistically significant (\(\\chi^2\) = 24.33, p < 0.001), moderate association (Cramer’s V = 0.321) between being ‘treated differently’ and ‘underrepresented’. Table [6](#tab%253AURvsTD) shows that those who don’t see themselves as treated differently have a higher than expected count for the ‘Not at all’ response to the underrepresented statement. Whereas, those who do regard themselves as being treated differently are more likely than expected to see themselves as underrepresented.

## Clinical Vs. Pre-clinical Students

### MCHS dimensions

Table 7: ANOVAs: testing differences between MCHS dimensions and clinic status (pre-clinical vs clicical students)

| Dimension | F | DF | p-val |  |
| --- | --- | --- | --- | --- |
| Open | 0.75 | 1 | 0.387 |  |
| Selfaware | 2.26 | 1 | 0.137 |  |
| Egoless | 14.91 | 1 | 0 *** |  |
| Suppinter | 0.07 | 1 | 0.786 |  |
| Selfrefl | 2.15 | 1 | 0.145 |  |
| *** p <= 0.001, ** p <= 0.01, * p <= 0.05 | | | | |

Comparing the MCHS dimensions between clinical and pre-clinical students, indicated that there was a statistically significant difference between these sets of students only for the ‘Egoless’ dimension, with nonclinical students scoring higher by 3.039 [1.475 - 4.604, 95% C.I.] (see table [7](#tab%253Aclinmchstab)).

### Added Likert items

Table 8: ANOVAs: testing differences between added likert items and clinic status (pre-clinical vs clicical students)

| Dimension | F | DF | p-val |  |
| --- | --- | --- | --- | --- |
| newQs1 | 0.02 | 1 | 0.892 |  |
| newQs2 | 0 | 1 | 0.95 |  |
| newQs3 | 2.79 | 1 | 0.098 |  |
| *** p <= 0.001, ** p <= 0.01, * p <= 0.05 | | | | |

Testing differences between scores on the added likert items and student’s clinical status indicated no significant differences on these items (see table [8](#tab%253AclinnewIstab))

Table 4: Underrepresented group identification Vs. Demographic Group: Chi-squared tests of association. P-values are estimated using a Monte-Carlo simulation with 5000 replicates. (Bsex - birth sex)

| Demographic group | Chi-squared | p-value |
| --- | --- | --- |
| Bsex | 1.612 | 0.663 |
| gender | 8.196 | 0.233 |
| ethnicity | 14.966 | 0.003 |
| disability | 16.257 | 0.003 |
| sexorient | 11.124 | 0.009 |
| religion | 10.856 | 0.089 |

Figure 3: Demographic factors effect on total MCHS score

Demographic factors effect on total MCHS score

|  | Estimate | Std. Error | t value | p value |
| --- | --- | --- | --- | --- |
| (Intercept) | 59.957 | 0.782 | 76.652 | 0.000 |
| genderMan | -0.046 | 0.938 | -0.049 | 0.961 |
| genderOther | 7.415 | 3.530 | 2.101 | 0.036 |
| ethnicityNon-white | 0.066 | 1.212 | 0.055 | 0.956 |
| disabilityYes | -6.944 | 1.499 | -4.634 | 0.000 |
| sexorientNon-hetero | 5.018 | 1.175 | 4.270 | 0.000 |
| religionChristn | -1.888 | 1.167 | -1.619 | 0.106 |
| religionOther | 0.052 | 1.185 | 0.044 | 0.965 |

### Health and Disability

| Types of Disability | Freq |
| --- | --- |
| Sensory impairments (such as those affecting sight or hearing) | 1 |
| Impairments with fluctuating or recurring effects (such as rheumatoid arthritis, myalgic encephalitis (ME), chronic fatigue syndrome (CFS), fibromyalgia, depression and epilepsy) | 2 |
| Progressive impairment (such as motor neurone disease, muscular dystrophy, and forms of dementia) | 1 |
| Auto-immune conditions (such as systemic lupus erythematosis) | 1 |
| Organ specific impairment, including respiratory conditions (such as asthma) and cardiovascular diseases (including thrombosis, stroke and heart disease) | 0 |
| Developmental impairment, such as autistic spectrum disorders (ASD), dyslexia and dyspraxia | 4 |
| Learning disabilities | 1 |
| Mental health conditions and illnesses | 5 |
| Impairment produced by injury to the body, including to the brain | 1 |

### Sexual Orientation

Which of the following best describes your sexual orientation?

Sexual orientation free text

| ResponseId | FreeText |
| --- | --- |
| R_2tnRYfX07XxmkaL | “I’m not sure. Maybe Queer?” |

### Religion

What is your religion?

Religion free text

| ResponseId | FreeText |
| --- | --- |
| R_10UeFyntZgPQ5zn | “undecided, all and none” |
| R_TdQNd65lgSoWWJP | “Quaker” |
| R_1IKGESN2zMEfhkK | “Christian - Orthodox” |

## Otherness

### Underrepresented Groups

To what extent do you feel you belong to/you are part of an underrepresented group in osteopathic education?

### Treated differently

Have you been treated differently based on your cultural background / identity?

How often have you been treated differently during training based on your cultural background / identity?

Treated differently free text

| ResponseId | FreeText |
| --- | --- |
| R_2QEfRR0HPaN3vMQ | “No” |
| R_2f8M4HRILFaSk84 | “Getting inappropriate comments towards physical appearance” |
| R_25vzGNF74UtAZ3n | “When I am not allowed to treat because of my gender” |
| R_3KAPYtyeiFbFiRE | “In the feedback I was referred to get a disability test done and not considering English is not my first language.” |
| R_TdQNd65lgSoWWJP | “I have once treated a non-binary patient in our student clinic, and because of my personal knowledge about LGBTQ+ issues, I ended up having to educate the clinical tutors about the physical health risks associated with chest binding. This helped me build my independence as a student practitioner but I have since had to spend time educating both peers and tutors which could have been done by staff who are knowledgable in these areas organising some lectures to help improve everyones education so that the student clinic can be a fully inclusive and accepting space.” |
| R_31YksGPVsKnkc4R | “Discrimination based on gender and age” |
| R_1hXWsOgmo6duC3E | “Clinic tutor said female students ask too many questions.” |
| R_0udLEVpq1HUHqRH | “As a foreign mature student I find it hard to find a partner to practice clinical skills with. This happens two to three times per week.” |
| R_1opNEOC7Kgpf6v9 | “Being examined by a tutor and her saying “due to her ethnicity she has large glutes”. Another tutor mentioning that I could call my black female patients “auntie” Being told my scrubs are too sexy because I am curvy even though everyone else wears them. Constantly being asked if my hair can be touched and where I am from. ” |
| R_1i41o07ov8Prr7G | “People avoiding me because I am not British” |
| R_29nskooLZgBfLI9 | “Condescending behaviour towards the working class” |
| R_3fDlxqpZPPhsIU8 | “I was not treated/spoken to fairly from a practice educator based on my gender and skin colour. Heteronormative and chauvinist bias between myself and another student - derived from gender and skin colour within UCO clinic.” |
| R_a9u8YOS1RwCGQAp | “No significant episode, on the outside, everything is always very correct and polite, but there sometimes is a whiff of”British superiority”, and you get a sense over time who supported Brexit and who did not, and that those who did would rather have it that you were not in the country. Which is ironic, with it being called “European School of Osteopathy”…” |
| R_7WmoHSR7TJs71T3 | “Tutor give me impression I’m stupid only because English is not my first language:/” |
| R_1DJ82luTS1HcKsw | “there are so many; the way they can relate diseases on black background, failing you, the look, avoiding touching you, lecture refusing to demonstrate technics on you, injustice, lying, they way they talk to you etc..” |

Who treated you differently

| Who | Freq |
| --- | --- |
| Academic staff | 7 |
| Clinic educator staff | 8 |
| Administration staff | 2 |
| Colleagues | 7 |
| Patients | 3 |
| Other | 1 |

Who treated you differently - free text

| ResponseId | FreeText |
| --- | --- |
| R_a9u8YOS1RwCGQAp | “Only some clinic admin staff (others are wonderful)” |
