# Supplementary material for: Overcoming barriers to equality, diversity, inclusivity, and sense of belonging in healthcare education: the Underrepresented Groups’ Experiences in Osteopathic Training (UrGEnT) mixed methods study
Source: BMC Med Educ. 2024 Apr 26;24:468. doi: 10.1186/s12909-024-05404-3 (PMC11055260; doi:10.1186/s12909-024-05404-3)
Supplement: Supplementary file 4 — Supplementary Material 4 [file 12909_2024_5404_MOESM4_ESM.docx]

# UrGEnT Quantitative Analysis

Author

Kevin Brownhill

Published

15 Nov, 2022. 7:15 am

## Introduction

There is a widely-identified need to address bullying, discrimination and harassment of under-represented groups in society. This also applies to students within educational institutions, and to patients within care settings. A recent systematic review of papers addressing these issues in undergraduate manual therapy education identified very little research specific to osteopathic educational institutions [[4](#ref-MacMillan2022)].

Cultural humility has been defined as the “ability to maintain an interpersonal stance that is other-oriented (or open to the other) in relation to aspects of cultural identity that are most important to the [person]” [[3](#ref-Hook2013)]. This emphasises an openness and humility on behalf of the healthcare practitioner when it comes to understanding another, who may have a different identity.

Gonzales et al [[2](#ref-Gonzalez2021)] developed and tested the Multidimensional Cultural Humility Scale (MCHS) on a sample of counsellors in the United States. They identified five dimensions of cultural humility using factor analysis: ‘Openness’, ‘Self-Awareness’, ‘Ego-less’, ‘Self-Reflection and Critique’, and ‘Supportive Interactions’.

This current analysis seeks to test whether the same factor structure applied to a sample of clinical and preclinical osteopathic students completing the MCHS, using confirmatory factor analysis (CFA).

## Lay summary

Due to the changes implemented to the survey, to adapt it to an osteopathic educational setting, and to preclinical and clinical stages of education, a factor analysis was conducted to see whether the 5-factor model, as proposed by Gonzales et al [[2](#ref-Gonzalez2021)] was adequate for this current data set. It was not possible to confirm or deny the adequacy of the model possibly due to the small sample size, a large number of missing values, non-normality of data, and possibly model misspecification.

An alternative model with less factors could be proposed, but we lack a theoretical justification for this. Recommendations for future studies are made.

## Methods

### Obtaining data

The target population was student osteopaths from UK OEIs. Responses were obtained via online questionnaire powered by Qualtrics (Qualtrics, Provo, UT).

#### Likert scale questions:

(six levels: from ‘strongly disagree’ to ‘strongly agree’, plus an added 7th category: ‘This has never crossed my mind’):

- Items of the MCHS - forward-coded
  - **open1**: ‘I am comfortable asking my patients about their cultural experience’
  - **open2**: ‘I seek to learn more about my patients’ cultural identity’
  - **open3**: ‘I believe that learning about my patients’ cultural background will allow me to better help my patients’
  - **selfaware1**: ‘I seek feedback from my clinic educators when working with diverse patients’
  - **selfaware2**: ‘I incorporate feedback I receive from colleagues and clinic educators when I am faced with problems regarding cultural interactions with patients’
  - **selfaware3**: ‘I am known by colleagues to seek advice when working with diverse patients’
  - **egoless1**: ‘I ask my patients about their cultural perspective on topics discussed during treatment sessions’
  - **egoless2**: ‘I ask my patients to describe their presenting problem based on their cultural background’
  - **egoless3**: ‘I ask my patients how they cope with problems in their culture’
  - **suppinter1**: ‘I enjoy learning from my weaknesses’
  - **suppinter2**: ‘I value feedback that improves my clinical skills’
  - **suppinter3**: ‘I evaluate my biases’
- Items of the MCHS - reverse-coded
  - **selfrefl1**: ‘I wait for others to ask about my biases for me to discuss them’
  - **selfrefl2**: ‘I do not necessarily need to resolve cultural conflicts with my patients during treatment sessions’
  - **selfrefl3**: ‘I believe the resolution of cultural conflict in treatment sessions is the patient’s responsibility’
- Three additional statements:
  - **newQs1**: ‘This topic is important to me’
  - **newQs2**: ‘I have the skills for asking patients about their backgrounds and experiences’
  - **newQs3**: ‘The clinical environment in my institution is appropriate to support asking about patients’ backgrounds and experiences’

#### Demographic questions:

- Whether a clinical or pre-clinical student
- Age
- Birth sex
- Gender
- Ethnicity
- Health and disability status
- Sexual orientation
- Religion

#### Other questions:

- ‘To what extent do you feel you belong to/you are part of an underrepresented group in osteopathic education?’
- ‘Have you been treated differently based on on your cultural background/identity?’
- ‘How often have you been treated differently during training based on your cultural background/identity?’
- ‘How often have you been treated differently during training based on your cultural background/identity?’
- ‘Please select to which group(s) the individual(s) who treated you differently came from’
- ‘Did you report the incident to your institution?’

### Statistical analysis

#### Likert variables

This section describes the results from the all the Likert scale questions. Figures below are centered onto a neutral response, and are ordered into MCHS forward-coded items ([Figure 1](#fig-liks1)), MCHS reverse-coded items ([Figure 2](#fig-liks2)), and new Likert variables ([Figure 3](#fig-liks3)). On the left of the figures are percentages of disagreement, agreement of the right. Bars are ordered by level of agreement.

Figure 1: Forward-coded MCHS variables.

Figure 2: Reverse-coded MCHS variables.

Figure 3: New Likert variables.

#### Data imputation

Missing data was imputed by means of ‘multivariate imputation by chained equations’, available from the R mice package [[7](#ref-vanBuuren2011)]. This predicts missing values using the multivariate dependence structure estimated from the available data. This is an iterative process, in which each variable’s missing data is imputed with a imputation model that can differ for each variable type, and is thus highly flexible. Each missing value is imputed and used in a subsequent iteration to update imputations for other missing values. Outputs from this process are monitored to ensure that a stable solution is obtained. In this case, 40 iterations were found to produce a stable set of imputed values. Predictive mean matching was used as the imputation model for the MCHS variables and ordinal variables, as it produced more realistic imputed values. Default imputation models were used for variables of other types.

Multiple imputation was used to provide multiple estimates of imputed values, more accurately reflecting the uncertainty in these estimates. In this case, 50 data sets were imputed. The distribution of the imputed values were compared against that of the observed variables to see if they were similar.

Model parameter estimates in subsequent analyses are based on pooled estimates, according to Rubin’s rule [[5](#ref-Rubin2004)].

#### Testing Normality

Multivariate normality testing was carried out using the Henze-Zirkler’s test, and by ploting univariate qq plots.

#### Confirmatory factor analysis

Confirmatory factor analysis was carried out to see if the MCHS data in the present study was adequately modelled by the 5-factor model proposed by [[2](#ref-Gonzalez2021)].

## Results

#### Missing data

[Figure 4](#fig-RF1) shows patterns of missing data. It is clear from this that there are large numbers of missing data points. Also, there appears to be a pattern whereby items encountered later in the questionnaire had greater numbers of missing data items (see [Figure 5](#fig-RF2)), suggesting a strong respondent fatigue effect.

Figure 4: Plot of missing data patterns per variable. Variables are arranged in order of number of number of missing values.

Figure 5: Order item encountered vs number of missing data points. Items encountered later tend to have more missing data.

#### Imputation

The distribution of imputed values were compared against the observed values (see [Figure 6](#fig-RF3)). This shows an acceptable correspondence between imputed and observed distributions.

Figure 6: Distribution of observed vs. imputed values (observed values shown in blue)

#### Checking normality assumption

Figure 7: Testing assumption of normality

Data was found to be non-normal: Henze-Zirkler statistic = 1.02, p-value = 0. See also [Figure 7](#fig-RF4). Therefore CFA was carried out using the variance-adjusted weighted least squares (WLSMV) estimator based on the polychoric correlation matrix (see [[6](#ref-Savalei2013)]).

#### CFA results

The results of fitting the 5-factor model of [[2](#ref-Gonzalez2021)] using the WLSMV estimator are shown in [Table 1](#tbl-convg.Lvar1), [Table 2](#tbl-mod_est.Lvar1), [Table 3](#tbl-mod_var.Lvar1) & [Table 4](#tbl-fitsMs.Lvar1). As can be seen from [Table 2](#tbl-mod_est.Lvar1) and [Table 3](#tbl-mod_var.Lvar1) there are improper solutions in the pooled estimates. This means these estimates cannot be trusted. Attempting to fix this by constraining negative variances to have a small positive value caused problems elsewhere (not shown). The model adequacy statistics indicate a good model fit by the usually adopted thresholds, but these are probably not valid when the WLSMV estimator is used [[8](#ref-Xia2019)].

Table 1: Convergence results and improper solutions for multiply imputed data using the WLSMV estimator.

| Converged | SE calculated | Heyward case - latent variables | Heyward case - observed variables |
| --- | --- | --- | --- |
| 45 | 45 | 0 | 12 |

Table 2: Pooled WLSMV estimator results: factor loadings. Estimates in red are improper solutions (completely standardised estimates > 1).

| Label | Estimate | S.E. | t | DF | p-value | Standardised |
| --- | --- | --- | --- | --- | --- | --- |
| Open =~ open1 | 1.00 | 0.00 |  |  |  | 0.61 |
| Open =~ open2 | 1.50 | 0.24 | 6.000 | 860 | 0.0e+00 | 0.90 |
| Open =~ open3 | 1.10 | 0.18 | 6.000 | 390 | 0.0e+00 | 0.65 |
| Selfaware =~ selfaware1 | 1.00 | 0.00 |  |  |  | 0.78 |
| Selfaware =~ selfaware2 | 0.93 | 0.18 | 5.200 | 200 | 4.0e-07 | 0.72 |
| Selfaware =~ selfaware3 | 0.76 | 0.15 | 5.100 | 210 | 8.0e-07 | 0.59 |
| Egoless =~ egoless1 | 1.00 | 0.00 |  |  |  | 0.79 |
| Egoless =~ egoless2 | 0.94 | 0.11 | 8.400 | 96 | 0.0e+00 | 0.74 |
| Egoless =~ egoless3 | 0.87 | 0.11 | 8.100 | 150 | 0.0e+00 | 0.69 |
| Selfrefl =~ selfrefl1 | 1.00 | 0.00 |  |  |  | 0.69 |
| Selfrefl =~ selfrefl2 | 1.40 | 0.42 | 3.200 | 140 | 1.5e-03 | 0.95 |
| Selfrefl =~ selfrefl3 | 0.74 | 0.24 | 3.100 | 140 | 2.4e-03 | 0.51 |
| Suppinter =~ suppinter1 | 1.00 | 0.00 |  |  |  | 0.20 |
| Suppinter =~ suppinter2 | 12.00 | 440.00 | 0.027 | Inf | 9.8e-01 | 2.40 |
| Suppinter =~ suppinter3 | 3.20 | 9.30 | 0.340 | 2700 | 7.3e-01 | 0.65 |

Table 3: Pooled WLSMV estimator results: variances and covariances. Estimates in red are improper solutions (variance estimates < 0).

| Label | Estimate | S.E. | t | DF | p-value | Standardised |
| --- | --- | --- | --- | --- | --- | --- |
| open1 ~~ open1 | 0.6300 | 0.000 |  |  |  | 0.630 |
| open2 ~~ open2 | 0.1900 | 0.000 |  |  |  | 0.190 |
| open3 ~~ open3 | 0.5800 | 0.000 |  |  |  | 0.580 |
| selfaware1 ~~ selfaware1 | 0.3900 | 0.000 |  |  |  | 0.390 |
| selfaware2 ~~ selfaware2 | 0.4900 | 0.000 |  |  |  | 0.490 |
| selfaware3 ~~ selfaware3 | 0.6500 | 0.000 |  |  |  | 0.650 |
| egoless1 ~~ egoless1 | 0.3800 | 0.000 |  |  |  | 0.380 |
| egoless2 ~~ egoless2 | 0.4600 | 0.000 |  |  |  | 0.460 |
| egoless3 ~~ egoless3 | 0.5400 | 0.000 |  |  |  | 0.540 |
| selfrefl1 ~~ selfrefl1 | 0.5200 | 0.000 |  |  |  | 0.520 |
| selfrefl2 ~~ selfrefl2 | 0.1900 | 0.000 |  |  |  | 0.190 |
| selfrefl3 ~~ selfrefl3 | 0.7600 | 0.000 |  |  |  | 0.760 |
| suppinter1 ~~ suppinter1 | 0.9600 | 0.000 |  |  |  | 0.960 |
| suppinter2 ~~ suppinter2 | 0.0180 | 0.000 |  |  |  | 0.018 |
| suppinter3 ~~ suppinter3 | 0.8200 | 0.000 |  |  |  | 0.820 |
| Open ~~ Open | 0.3700 | 0.098 | 3.80 | 850 | 1.5e-04 | 1.000 |
| Selfaware ~~ Selfaware | 0.6100 | 0.130 | 4.60 | 230 | 6.8e-06 | 1.000 |
| Egoless ~~ Egoless | 0.6200 | 0.098 | 6.30 | 140 | 0.0e+00 | 1.000 |
| Selfrefl ~~ Selfrefl | 0.4800 | 0.190 | 2.60 | 130 | 1.2e-02 | 1.000 |
| Suppinter ~~ Suppinter | 0.0410 | 0.054 | 0.76 | 150 | 4.5e-01 | 1.000 |
| Open ~~ Selfaware | 0.2100 | 0.054 | 3.90 | 1100 | 1.2e-04 | 0.430 |
| Open ~~ Egoless | 0.2900 | 0.066 | 4.30 | 180 | 2.4e-05 | 0.600 |
| Open ~~ Selfrefl | 0.0450 | 0.063 | 0.71 | 910 | 4.8e-01 | 0.110 |
| Open ~~ Suppinter | -0.0270 | 0.028 | -0.97 | 150 | 3.3e-01 | -0.210 |
| Selfaware ~~ Egoless | 0.0830 | 0.069 | 1.20 | 210 | 2.3e-01 | 0.140 |
| Selfaware ~~ Selfrefl | 0.0390 | 0.080 | 0.49 | 460 | 6.2e-01 | 0.073 |
| Selfaware ~~ Suppinter | -0.0460 | 0.040 | -1.10 | 100 | 2.6e-01 | -0.290 |
| Egoless ~~ Selfrefl | -0.0390 | 0.080 | -0.49 | 330 | 6.2e-01 | -0.072 |
| Egoless ~~ Suppinter | 0.0033 | 0.025 | 0.13 | 200 | 8.9e-01 | 0.021 |
| Selfrefl ~~ Suppinter | -0.0460 | 0.046 | -1.00 | 220 | 3.1e-01 | -0.330 |

Table 4: Model adequacy statistics for pooled WLSMV estimator results. chisq.scaled = scaled model Chi-squared, cfi.scaled = scaled comparative fit index, tli.scaled = scaled Tucker-Lewis Index, rmsea.scaled = scaled root mean square error of approximation

|  | Statistic | DF | p-value |
| --- | --- | --- | --- |
| chisq.scaled | 94.27 | 80 | 0.13 |
| cfi.scaled | 0.94 |  |  |
| tli.scaled | 0.93 |  |  |
| rmsea.scaled | 0.04 |  | 0.80 |

## Conclusion

It was not possible to confirm or deny the adequacy of the 5-factor model, as proposed by Gonzales et al [[2](#ref-Gonzalez2021)], for the current data set, due to the presence of improper solutions in the pooled model estimates. Improper solutions are the result of a one of: small sample size, a large number of missing values, non-normality of data, and possibly model misspecification [[1](#ref-Chen2001)]. There is possibly all four in this data set. It may be possible to propose a different model with a fewer number of factors, which may be more estimable given the small sample size and the small number of items per factor. However, we do not currently have a theoretical justification for a model with fewer factors, and not having enough data is probably not a good justification for this. Future studies would require a greater sample size, possibly randomisation of the order of the items so that missing data does not load so heavily on later items, and reducing the overall respondent burden of the survey. Future studies should perhaps not include the ‘This has never crossed my mind’ option, as this also lead to more missing data, as it is not part of the scale. Instead a neutral level could be added instead: ‘Neither agree or disagree’.

## References

[1]

F. Chen, K.A. Bollen, P. Paxton, P.J. Curran, J.B. Kirby, Improper solutions in structural equation models: Causes, consequences, and strategies, Sociological Methods & Research. 29 (2001) 468–508.

[2]

E. Gonzalez, K.R. Sperandio, P.R. Mullen, V.E. Tuazon, Development and initial testing of the multidimensional cultural humility scale, Measurement and Evaluation in Counseling and Development. 54 (2021) 56–70.

[3]

J.N. Hook, D.E. Davis, J. Owen, E.L. Worthington Jr, S.O. Utsey, Cultural humility: Measuring openness to culturally diverse clients., Journal of Counseling Psychology. 60 (2013) 353.

[4]

A. MacMillan, D. Hohenschurz-Schmidt, V. Migliarini, J. Draper-Rodi, Discrimination, bullying or harassment in undergraduate education in the osteopathic, chiropractic and physiotherapy professions: A systematic review with critical interpretive synthesis, International Journal of Educational Research Open. 3 (2022) 100105.

[5]

D.B. Rubin, Multiple imputation for nonresponse in surveys, John Wiley & Sons, 2004.

[6]

V. Savalei, M. Rhemtulla, The performance of robust test statistics with categorical data, British Journal of Mathematical and Statistical Psychology. 66 (2013) 201–223.

[7]

S. van Buuren, K. Groothuis-Oudshoorn, [mice: Multivariate imputation by chained equations in r](https://doi.org/10.18637/jss.v045.i03), Journal of Statistical Software. 45 (2011) 1–67.

[8]

Y. Xia, Y. Yang, RMSEA, CFI, and TLI in structural equation modeling with ordered categorical data: The story they tell depends on the estimation methods, Behavior Research Methods. 51 (2019) 409–428.
